# Hydroxychloroquine in recurrent pregnancy loss: data from a French prospective multicenter registry

**DOI:** 10.1093/humrep/deae146

**Published:** 2024-06-28

**Authors:** Amandine Dernoncourt, Kaies Hedhli, Noémie Abisror, Meryam Cheloufi, Jonathan Cohen, Kamila Kolanska, Chloé McAvoy, Lise Selleret, Eric Ballot, Emmanuelle Mathieu d’Argent, Nathalie Chabbert Buffet, Olivier Fain, Gilles Kayem, Arsène Mekinian

**Affiliations:** Service de Médecine Interne et RECIF, Centre Hospitalo-Universitaire Amiens-Picardie, Amiens, France; Laboratoire d’Hématologie, Centre de Biologie Humaine, Centre Hospitalo-Universitaire Amiens-Picardie, Amiens, France; Service de Médecine Interne et Inflammation-Immunopathology-Biotherapy Department (DMU I3), Hôpital Saint Antoine, Sorbonne Université AP-HP, Paris, France; Service de Gynécologie Obstétrique, Hôpital Armand-Trousseau, Sorbonne Université AP-HP, Paris, France; Service d’Obstétrique et de Fertilité, Clinique Saint Thérèse, Paris, France; Service de Gynécologie Obstétrique et Médecine de la Reproduction, Centre de Fertilité Tenon, Hôpital Tenon, Sorbonne Université AP-HP, Paris, France; Service de Médecine Interne et Inflammation-Immunopathology-Biotherapy Department (DMU I3), Hôpital Saint Antoine, Sorbonne Université AP-HP, Paris, France; Service de Gynécologie Obstétrique et Médecine de la Reproduction, Centre de Fertilité Tenon, Hôpital Tenon, Sorbonne Université AP-HP, Paris, France; Service d’Immunologie, Hôpital Saint Antoine, Sorbonne Université AP-HP, Paris, France; Service de Gynécologie Obstétrique et Médecine de la Reproduction, Centre de Fertilité Tenon, Hôpital Tenon, Sorbonne Université AP-HP, Paris, France; Service de Gynécologie Obstétrique et Médecine de la Reproduction, Centre de Fertilité Tenon, Hôpital Tenon, Sorbonne Université AP-HP, Paris, France; Service de Médecine Interne et Inflammation-Immunopathology-Biotherapy Department (DMU I3), Hôpital Saint Antoine, Sorbonne Université AP-HP, Paris, France; Service de Gynécologie Obstétrique, Hôpital Armand-Trousseau, Sorbonne Université AP-HP, Paris, France; Service de Médecine Interne et Inflammation-Immunopathology-Biotherapy Department (DMU I3), Hôpital Saint Antoine, Sorbonne Université AP-HP, Paris, France

**Keywords:** hydroxychloroquine, recurrent pregnancy loss, pregnancy complications, spontaneous abortion, treatment outcome

## Abstract

**STUDY QUESTION:**

What are the outcomes of pregnancies exposed to hydroxychloroquine (HCQ) in women with a history of recurrent pregnancy loss (RPL), and what factors predict the course of these pregnancies beyond the first trimester?

**SUMMARY ANSWER:**

In our cohort of pregnancies in women with a history of RPL exposed to HCQ early in pregnancy, we found that the only factor determining the success of these pregnancies was the number of previous miscarriages.

**WHAT IS KNOWN ALREADY:**

Dysregulation of the maternal immune system plays a role in RPL. HCQ, with its dual immunomodulating and vascular protective effects, is a potential treatment for unexplained RPL.

**STUDY DESIGN, SIZE, DURATION:**

The FALCO (Facteurs de récidive précoce des fausses couches) registry is an ongoing French multicenter infertility registry established in 2017 that includes women (aged from 18 to 49 years) with a history of spontaneous RPL (at least three early miscarriages (≤12 weeks of gestation (WG)) recruited from several university hospitals.

**PARTICIPANTS/MATERIALS, SETTING, METHODS:**

Spontaneous pregnancies enrolled in the FALCO registry with an exposure to HCQ (before conception or at the start of pregnancy) were included. Pregnancies concomitantly exposed to tumor necrosis factor inhibitors, interleukin-1 and -2 inhibitors, intravenous immunoglobulin, and/or intravenous intralipid infusion, were excluded. Concomitant treatment with low-dose aspirin (LDA), low-molecular weight heparin (LMWH), progesterone, and/or prednisone was allowed. All patients underwent the recommended evaluations for investigating RPL. Those who became pregnant received obstetric care in accordance with French recommendations and were followed prospectively. The main endpoint was the occurrence of a pregnancy continuing beyond 12 WG, and the secondary endpoint was the occurrence of a live birth.

**MAIN RESULTS AND THE ROLE OF CHANCE:**

One hundred pregnancies with HCQ exposure in 74 women were assessed. The mean age of the women was 34.2 years, and the median number of previous miscarriages was 5. Concomitant exposure was reported in 78 (78%) pregnancies for prednisone, 56 (56%) pregnancies for LDA, and 41 (41%) pregnancies for LMWH. Sixty-two (62%) pregnancies ended within 12 WG, the other 38 (38%) continuing beyond 12 WG. The risk of experiencing an additional early spontaneous miscarriage increased with the number of previous miscarriages, but not with age. The distributions of anomalies identified in RPL investigations and of exposure to other drugs were similar between pregnancies lasting ≤12 WG and those continuing beyond 12WG. The incidence of pregnancies progressing beyond 12 WG was not higher among pregnancies with at least one positive autoantibody (Ab) (i.e. antinuclear Ab titer ≥1:160, ≥1 positive conventional and/or non-conventional antiphospholipid Ab, and/or positive results for ≥1 antithyroid Ab) without diminished ovarian reserve (18/51, 35.3%) than among those without such autoantibody (18/45, 40.0%) (*P *=* *0.63). Multivariate analysis showed that having ≤4 prior miscarriages was the only factor significantly predictive for achieving a pregnancy > 12 WG, after adjustment for age and duration of HCQ use prior to conception (adjusted odds ratio (OR) = 3.13 [1.31–7.83], *P *=* *0.01).

**LIMITATIONS, REASONS FOR CAUTION:**

Our study has limitations, including the absence of a control group, incomplete data for the diagnostic procedure for RPL in some patients, and the unavailability of results from endometrial biopsies, as well as information about paternal age and behavioral factors. Consequently, not all potential confounding factors could be considered.

**WIDER IMPLICATIONS OF THE FINDINGS:**

Exposure to HCQ in early pregnancy for women with a history of RPL does not seem to prevent further miscarriages, suggesting limited impact on mechanisms related to the maternal immune system.

**STUDY FUNDING/COMPETING INTEREST(S):**

The research received no specific funding, and the authors declare no competing interests.

**TRIAL REGISTRATION NUMBER:**

clinicaltrial.gov NCT05557201.

## Introduction

In human pregnancies, gestational failures generally occur at early stages, and are mostly due to a failure of the embryo to implant. Implantation is a key step in human reproduction dependent on intricate interactions between a competent embryo and the maternal uterine endometrium, which must be both receptive and selective (Dimitriadis *et al.*, 2020; Colamatteo *et al.*, 2023). Diverse genetic, anatomic, hormonal, hematological, and immunological factors underlie these processes, the disruption of any of which can lead to pregnancy loss (Dimitriadis *et al.*, 2020; Colamatteo *et al.*, 2023).

Pregnancy loss, also known as *miscarriage*, is defined as the spontaneous abortion of a pregnancy before the fetus reaches viability. Recurrent pregnancy loss (RPL) is highly challenging to diagnose and treat, with about half of all cases remaining unexplained, and no consensus on effective therapies (Dimitriadis *et al.*, 2020; Quenby *et al.*, 2021; ESHRE Guideline Group on RPL *et al.*, 2023).

Advanced maternal age and the number of previous pregnancy losses are both well-established risk factors for RPL (Lund *et al.*, 2012; Magnus *et al.*, 2019). Indeed, embryonic chromosomal aberrations are a major cause of miscarriage, and the risk of early pregnancy loss due to fetal aneuploidy increases as the mother ages (Franasiak *et al.*, 2014; Dimitriadis *et al.*, 2020; Quenby *et al.*, 2021; ESHRE Guideline Group on RPL *et al.*, 2023).

In some cases, RPL seems to be associated with immune imbalances in the endometrium, characterized by an excessive inflammatory response harmful to embryo development, and a deficiency of anti-inflammatory and regulatory responses essential for maternal immunotolerance to fetal alloantigens (Dimitriadis *et al.*, 2020; Zhao *et al.*, 2021; Colamatteo *et al.*, 2023). These local immunological disorders have been identified in unexplained RPL, in women with uterine tissue disorders, such as endometriosis, and in those with systemic disorders, such as obesity and polycystic ovary syndrome (Dimitriadis *et al.*, 2020; Pirtea *et al.*, 2021). Immunomodulatory therapies are, therefore, currently being investigated as a potential treatment option for RPL (Mekinian *et al.*, 2016; Cavalcante *et al.*, 2023).

Hydroxychloroquine (HCQ), initially developed as an antimalarial drug, has been widely used as a disease-modifying antirheumatic drug for the treatment of rheumatic auto-immune diseases, including systemic lupus erythematosus in particular (Schrezenmeier and Dörner, 2020). HCQ has direct and indirect immunomodulatory effects, and can inhibit specific cellular functions and molecular pathways involved in both innate and adaptive immune responses; it may, therefore, be effective against some of the mechanisms underlying unexplained RPL (de Moreuil *et al.*, 2020; Schrezenmeier and Dörner, 2020). Specifically, HCQ inhibits antigen processing and presentation, subsequent T-cell activation, and the production of pro-inflammatory cytokines (Schrezenmeier and Dörner, 2020). It also has vascular protective effects through the reduction of both endothelial dysfunction and hypercoagulability, particularly through the inhibition of antiphospholipid antibody (aPL) binding and platelet aggregation (de Moreuil *et al.*, 2020; Hoxha *et al.*, 2022). In the context of recurrent miscarriages, HCQ might be able to prevent an overactive pro-inflammatory response at the maternal–fetal interface, and might promote the vascular remodeling of spiral arteries crucial for placental development. However, there are no published clinical data concerning the potential benefits of HCQ for achieving live births in patients with RPL.

In this study, we assessed the outcomes of pregnancies in women with a history of RPL exposed to HCQ before conception or early in gestation, with the aim of identifying potential predictive factors that could be acted on to promote the progression of pregnancies beyond the first trimester.

## Materials and methods

### Study design

The FALCO (Facteurs de récidive précoce des fausses couches) registry is a French multicenter observational infertility registry established in 2017 (clinicaltrial.gov NCT05557201) that includes women (aged from 18 to 49 years) with a history of unexplained spontaneous RPL (at least three early miscarriages (≤12 weeks of gestation (WG)), consecutive or not, with the same partner) recruited from several university hospitals (Saint-Antoine Hospital (Paris, France), Tenon Hospital (Paris, France) and Trousseau Hospital (Paris, France)). The patients underwent the recommended evaluations for investigating RPL, and those meeting antiphospholipid syndrome (APS) classification criteria were not enrolled in the registry (Miyakis *et al.*, 2006; Barbhaiya *et al.*, 2023). Those who became pregnant received obstetric care in accordance with French recommendations and were followed prospectively.

### Population study

The eligibility criteria for this analysis were: spontaneous pregnancies in women enrolled in the FALCO registry and exposed to HCQ either before conception or at the start of pregnancy. Pregnancies in patients on long-term treatment with HCQ for autoimmune diseases (particularly systemic lupus erythematosus), and those concomitantly exposed to tumor necrosis factor inhibitors, interleukin-1 and -2 inhibitors, intravenous immunoglobulin, and/or intravenous intralipid infusion, were excluded. Pregnancies in patients with chronic autoimmune disease not treated with these therapies, including HCQ, were not excluded. Concomitant treatment with low-dose aspirin (LDA), low-molecular weight heparin (LMWH), progesterone, and/or prednisone was allowed.

### Data collection and RPL diagnosis

For pregnancies with available data, we collected demographic and clinical characteristics (age, body mass index), lifestyle habits (tobacco, alcohol use, coffee consumption during pregnancy), underlying medical conditions, obstetric history (number of previous miscarriages, history of late miscarriage (13–20 WG), stillbirth (>20 WG) and/or livebirth), gynecological disorders (polycystic ovary syndrome, endometriosis, or adenomyosis), and concomitant medication.

Likewise, we collected available results of tests conducted to investigate RPL. The diagnostic work-up recommended by ESHRE is as follows (ESHRE Guideline Group on RPL *et al.*, 2023): uterine cavity assessment by hysteroscopy or hysterosonography, and screening for conventional aPL (lupus anticoagulant (LA), anticardiolipin antibodies (Abs) (aCL), and anti-β2 glycoprotein I Abs (aβ2GPI)), thyroid function (thyroid-stimulating hormone (TSH) levels, and antithyroid Abs (i.e. antithyroid peroxidase and antithyroglobulin Abs)). Additionally, other tests were performed, including the determination of ovarian reserve parameters (most recent antral follicular count, and anti-Mullerian hormone (AMH) level), parental chromosomal karyotypes, antinuclear Abs (ANA), screening for non-conventional aPL (anti-phosphatidylserine/prothrombin, anti-phosphatidylethanolamine, and anti-annexin V Abs), and semen analysis for the woman’s partner.

Finally, pregnancy outcomes were recorded.

### Endpoints

The main endpoint was the occurrence of a pregnancy continuing beyond 12 WG, and the secondary endpoint was the occurrence of a live birth.

### Statistical analysis

In the descriptive analysis, categorical variables are presented as numbers (percentages), and continuous variables are presented as mean ± SD or median (interquartile range (IQR)), according to the data distribution. The Shapiro–Wilk test was used to determine whether data followed a normal distribution.

For bivariate analyses comparing pregnancies of >12 WG with ≤12 WG, continuous variables were compared in Student’s *t*-tests or Mann–Whitney *U* tests, depending on the data distribution. Categorical variables were compared in chi-squared or Fisher’s exact test, as appropriate. The Kruskal–Wallis test was used as a non-parametric method for multiple group comparisons across different age groups.

Multivariate analysis by logistic regression model was conducted to identify independent factors predictive of pregnancy progression beyond 12 WG. Variables from the collected dataset were included in the model, with criteria ensuring less than 20% of patients with missing data or variables with less than 5% missing values. The adjustment variables were defined *a priori* based on literature data (i.e. maternal age and duration of HCQ use) (Munster *et al.*, 2002; Magnus *et al.*, 2019; Dima *et al.*, 2022). Additional potential adjustment variables were evaluated using a penalized Least Absolute Shrinkage and Selection Operator (LASSO) regression model, with the penalty coefficient (lambda) selected to minimize estimation error while maintaining model parsimony (Tibshirani, 1996). Multicollinearity was investigated using the variance inflation factor (O’Brien, 2007), and variables of the best-performing algorithm were selected through step-wise use of Akaike’s information criterion and Schwarz’s Bayesian criterion (Burnham and Anderson, 2004). Results for independent predictive factors are reported as odds ratios (ORs) with 95% CI.

Values of *P *<* *0.05 were considered statistically significant.

All analyses were performed with Addinsoft XLSTAT version 2023.2.0 software.

### Ethics

The study was conducted in compliance with French legislation and the Declaration of Helsinki as concerns ethics principles for medical research involving human subjects. The local ethics committee (CPP Ile de France VIII) approved the study protocol on 9 January 2024.

## Results

### Population study

**Figure 1.**
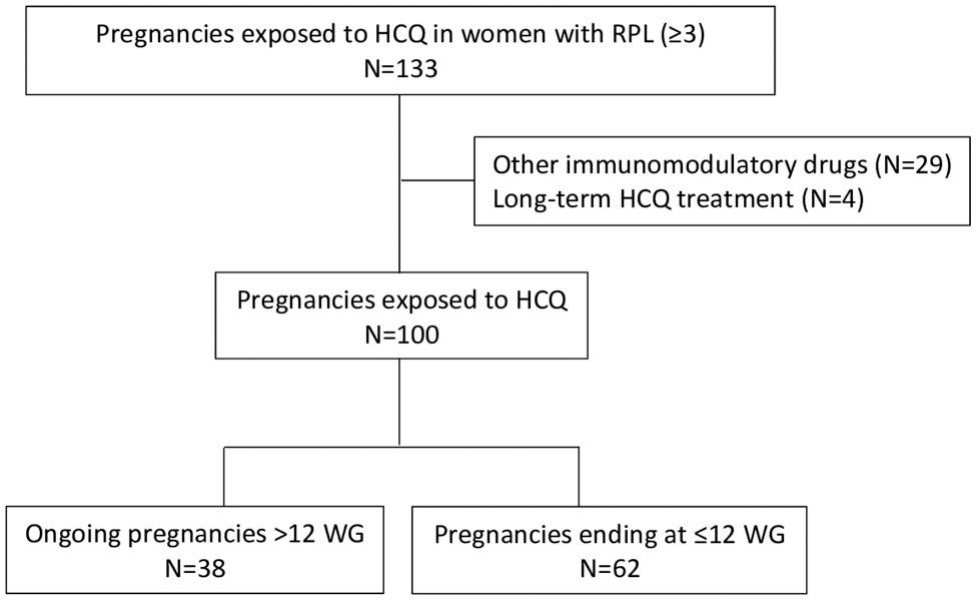
**Study population.** HCQ, hydroxychloroquine; RPL, recurrent pregnancy loss; WG, week of gestation.

This study included 100 pregnancies exposed to HCQ in a cohort of 74 women (Fig. 1). The baseline characteristics of the study population are detailed in Table 1. The mean age at pregnancy was 34.2 (±4.7) years, and 43 (43%) pregnancies had a previous live birth. Seven (7%) women had a medical history of autoimmune diseases, and all were organ-specific: four with Hashimoto’s thyroiditis, one with ulcerative colitis, and two with celiac disease. The median number of previous miscarriages was 5 [IQR 2], and the maximum number was 12, with no significant difference in the number of previous miscarriages between the various age groups (*P *=* *0.99).

**Table 1.** Comparative analysis of pregnancies continuing beyond 12 WG and pregnancies ending by 12 WG.

	HCQ pregnancies			*P*-value
	Total	Ongoing pregnancies (>12 WG)	Lost pregnancies (≤12) WG	
	(N* *=* *100)	(N* *=* *38)	(N* *=* *62)	
**Demographic and clinical characteristics**				
Age at pregnancy (years)				
• Mean	34.2 (±4.7)	34.8 (±4.0)	33.9 (±5.2)	0.38
• ≤29	21/100 (21.0)	5/38 (13.2)	16/62 (25.8)	0.21
• 30–34	28/100 (28.0)	12/38 (31.6)	16/62 (25.8)	0.70
• 35–39	41/100 (41.0)	18/38 (47.4)	23/62 (37.1)	0.42
• ≥40	10/100 (10.0)	3/18 (7.9)	7/62 (11.3)	0.74
Active smokers	22/79 (27.8)	5/25 (40.0)	17/54 (31.5)	0.42
Alcohol use	0/79			
High levels of caffeine consumption (>300 mg/d)	10/33 (30.3)	5/11 (45.5)	5/22 (22.7)	0.24
Obesity (BMI ≥ 30 kg/m^2^)	9/86 (10.5)	1/26 (3.8)	8/60 (13.3)	0.25
Diabetes	0/100			
Autoimmune diseases	7/100* (7.0)	4/38 (10.5)	3/62 (4.8)	0.42
**Obstetric history**				
Nulliparity	54/100 (54.0)	18/38 (47.4)	36/62 (58.1)	0.40
Number of previous miscarriages				
• Median	5 [2]	4 [1.5]	5 [2]	**0.002**
• 3	15/100 (15.0)	10/38 (26.4)	5/62 (8.1)	**0.02**
• 4	27/100 (27.0)	12/38 (31.6)	15/62 (24.2)	0.45
• 5	26/100 (26.0)	9/38 (23.7)	17/62 (27.4)	0.42
• 6	18/100 (18.0)	6/38 (15.8)	12/62 (19.4)	0.65
• ≥7	14/100 (14.0	1/38 (2.6)	13/62 (9.7)	**0.02**
History of late miscarriage	17/100 (17.0)	7/38 (18.4)	10/62 (16.1)	0.98
History of stillbirth	6/100 (6.0)	4/38 (10.5)	2/62 (3.2)	0.20
History of live birth	43/100 (43.0)	18/38 (47.4)	25/62 (40.3)	0.63
**Test results**				
PCOS	10/100 (10.0)	3/38 (7.9)	7/62 (11.3)	0.73
Endometriosis or adenomyosis	21/100 (21.0)	9/38 (23.7)	12/62 (19.3)	0.61
Uterine cavity abnormalities	7/100 (7.0)	5/37 (13.5)	2/61 (3.3)	0.10
DOR (AFC ≤4 and AMH <1.0 ng/mL)	3/64 (4.7)	1/19 (5.3)	2/45 (4.4)	1.0
≥1 positive conventional aPL	1/99 (1.0)	1/37 (2.7)	0/62	NA
≥1 positive non-conventional aPL	12/79 (15.2)	2/26 (7.7)	10/53 (18.9)	0.32
Positive ANA (titer ≥1:160)	36/96 (37.5)	12/36 (33.3)	24/60 (40.0)	0.66
≥1 positive antithyroid Ab	14/87 (16.1)	7/34 (20.6)	7/53 (13.2)	0.55
Abnormal TSH	1/80 (1.3)	0/29	1/51 (2.0)	NA
Parental abnormal chromosomal karyotype	5/95 (5.3)	0/36	5/59 (8.5)	0.15
High sperm DNA fragmentation index (≥20%)	5/54 (9.3)	1/19 (5.3)	4/35 (11.4)	0.65
**Treatments**				
Initiation of HCQ prior to conception	71/100 (71.0)	27/38 (71.1%)	44/62 (71.0%)	0.99
• Duration of HCQ use (weeks), median	8.7 [8.6]	4.4 [4.3] (n* *=* *25)	9 [9] (n* *=* *43)	0.20
Initiation of HCQ in early gestation (<6 WG)	29/100 (29.0)	11/38 (28.9%)	18/62 (29.0%)	0.99
Prophylactic LMWH	41/100 (41.0)	14/38 (36.8)	27/62 (43.5)	0.65
Low-dose aspirin	56/100 (56.0)	20/38 (52.6)	36/62 (58.1)	0.75
Progesterone	35/100 (35.0)	15/38 (39.5)	20/62 (32.3)	0.61
Prednisone				
• Use	78/100 (78.0)	31/38 (81.6)	47/62 (75.8)	0.66
• Dose (mg/d), median	10 [0] (n* *=* *75)	10 [0] (n* *=* *31)	10 [0] (n* *=* *44)	1.0
**Pregnancy outcomes**				
Pregnancies ending at ≤12 WG	62/100 (62.0)			
Pregnancies continuing after 12WG	38/100 (38.0)			
• Singleton pregnancies	35/35 (100.0)			
• Late miscarriages (13–20 WG)	4/99** (4.0)			
• Stillbirths (>20 WG)	2/99** (2.0)			
• Live births	31/99** (31.3)			

The data shown are the mean (±SD), median [interquartile range], n, or n*/*N (%).

* Four cases with Hashimoto’s thyroiditis, one with ulcerative colitis, and two with celiac disease.

** One pregnancy was lost to follow-up beyond 12 WG.

ANA, antinuclear antibodies; AFC, antral follicle count; AMH, anti-Müllerian hormone; Abs, antibodies; aPL, antiphospholipid antibodies; DOR, diminished ovarian reserve; HCQ, hydroxychloroquine; LMWH, low-molecular weight heparin; WG, week of gestation.

### Investigations for RPL

The results of the initial assessment for RPL are presented in Table 1. For 76 (76%) pregnancies, RPL remained unexplained after the recommended diagnostic workup by ESHRE. In 34 (34%) pregnancies, at least one risk factor was identified but the patients had already undergone specific treatments, including surgery for septal defects or thyroid replacement therapy for hypothyroidism. Only one woman tested positive for aCL on two tests performed more than 12 weeks apart, without the presence of aβ2GPI or LA, and did not meet the full criteria for APS. Non-conventional aPL were found in 12 of 79 cases (15.2%). Only one of the patients with endometriosis had severe deep endometriosis. At least one positive result for an autoantibody (i.e. titer ANA ≥1:160, ≥1 positive result for conventional or non-conventional aPL, and/or positive results for ≥1 antithyroid Ab) was obtained in 53 of 99 (53.5%) pregnancies, including 51 (51.5%) pregnancies without diminished ovarian reserve.

### Treatment

In all the pregnancies included, HCQ was used at a dose of 400 mg per day. For 71 (71%) pregnancies, HCQ treatment began before conception, and the median duration of exposure prior to pregnancy was 8.7 [IQR 8.6] weeks. In the other 29 pregnancies (29%), it was initiated early in gestation, once pregnancy was confirmed by blood or urine test, and before 6 WG. Concomitant exposure to prednisone was reported in 78 (78%) pregnancies, with a median dosage of 10 [IQR 0] mg per day, to LDA in 56 (56%) pregnancies, and to prophylactic anticoagulation with LMWH in 41 (41%) pregnancies (Table 1).

Regarding aPL-positive pregnancies (n = 13), two (15.4%) were on LDA, five (38.5%) (including the aCL-pregnancy) on LMWH, two (15.4%) on both LMWH and LDA, and four (30.7%) on neither.

### Pregnancy outcomes

Sixty-two (62%) pregnancies ended within 12 WG, with a median term of 9 [IQR 3] weeks, whereas 38 (38%) continued beyond 12 WG. Finally, 31 of 99 (31.3%, data not available for one pregnancy) resulted in live births (Table 1).

**Figure 2.**
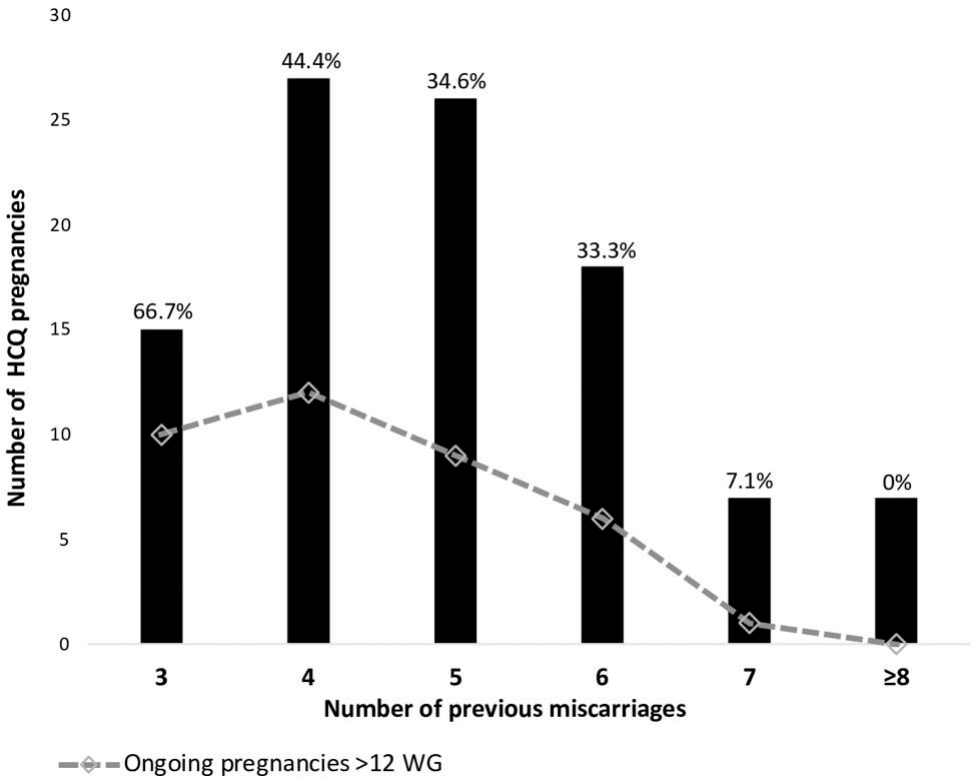
**Incidence of ongoing pregnancies beyond 12 weeks of gestation according to the number of previous miscarriages.** The bars indicate the number of pregnancies exposed to HCQ, and the dots indicate the number of these pregnancies ongoing beyond 12 weeks, according to the number of previous miscarriages. HCQ, hydroxychloroquine; WG, week of gestation.

The incidence of pregnancies progressing beyond 12 WG decreased with increasing number of previous miscarriages (Fig. 2). Only one (7.1%) of the pregnancies in women with at least seven previous miscarriages (n* *=* *14), continued beyond 12 WG on HCQ and resulted in a live birth (Fig. 2).

### Comparison of pregnancies with durations of >12 WG and ≤12 WG

Mean age at the time of pregnancy did not differ significantly between the two groups. Similarly, no significant associations were found with lifestyle habits or underlying conditions (Table 1). The progression of pregnancy beyond 12 WG was, notably, associated with a smaller number of previous miscarriages. A higher proportion of pregnancies that ended ≤12 WG had experienced >4 previous miscarriages (42/62, 67.7%) in comparison with pregnancies >12 WG (16/38, 42.1%) (*P *=* *0.018). A history of previous live birth did not seem to influence the likelihood of achieving a successful pregnancy. Similarly, the distribution of anomalies identified in RPL investigations did not differ significantly between the two groups (Table 1). Cytogenetic analysis of product of conception (POC) was performed on a subset of six of 62 (9.7%) early miscarriages, and chromosomal abnormalities were detected in only one (3.3%) case. The incidence of pregnancies progressing beyond 12 WG was not higher among pregnancies with a positive autoantibody result without diminished ovarian reserve (18/51, 35.3%) than those without autoantibodies (18/45, 40.0%) (*P *=* *0.63).

The occurrence of a successful pregnancy >12WG did not differ between pregnancies exposed to HCQ prior to conception (27/71, 38.0%) and those exposed after the onset of pregnancy (11/29, 37.9%) (*P *=* *1.0). Among pregnancies exposed to HCQ before conception with a known start date (n = 68), 47 (69.1%) were exposed for ≤3 months, and 21 (30.9%) for >3 months, with a similar risk of experiencing an additional early miscarriage between both groups (28/47, 59.6% vs 15/21, 71.4% respectively) (*P *=* *0.49). Only six pregnancies were exposed to HCQ for >6 months in pre-conception, of which two (33.3%) progressed beyond the first trimester.

HCQ was initially prescribed until the end of the first trimester, and it was subsequently discontinued in the event of miscarriage in all pregnancies ≤12 WG. For most pregnancies >12 WG, HCQ was stopped at the end of the first trimester in 25 of 35 (71.4%) cases as planned. However, in 10 (28.6%) pregnancies >12 WG, HCQ was continued beyond the first trimester, and its use was prolonged until delivery in 6 of the 31 (19.3%) live births.

Concomitant exposure to other drugs was similar in the two groups (Table 1). The incidence of pregnancies progressing beyond 12 WG in women treated with HCQ but not exposed to prednisone was 31.8% (7/22), whereas it was 39.7% (31/78) in those treated with prednisone (*P *=* *0.50), with a live birth rate of 28.6% (6/21) versus 32.1% (25/78), respectively (*P *=* *0.76).

Multivariate analysis showed that having ≤4 prior miscarriages was the only factor significantly associated with pregnancy continuing beyond 12 WG, after adjustment for age and duration of HCQ use prior to conception (adjusted OR = 3.13 [1.31–7.83], *P *=* *0.01) (Table 2).

**Table 2.** Predictive factors for pregnancy progressing beyond 12 WG based on logistic regression analysis.

	Unadjusted OR [95% CI], *P*-value	Adjusted OR [95% CI]*, *P*-value
Number of previous miscarriages		
=3	4.07 [1.32–14.10], *P *=* *0.02	4.52 [1.28–18.70], *P *=* *0.02
≤4	2.89 [1.26–6.77], *P *=* *0.01	3.13 [1.31–7.83], *P *=* *0.01
>4	0.34 [0.15–0.80], *P *=* *0.01	0.32[0.13–0.76], *P *=* *0.01

* Adjusted for maternal age and duration of HCQ use prior to conception (post-conception exposure was set at 0 weeks).

The variables included in the analysis were: maternal age at pregnancy, history of late miscarriage and/or stillbirth, history of live birth, polycystic ovary syndrome, endometriosis or adenomyosis, positive antinuclear Ab, positive antithyroid Ab, HCQ initiation prior to conception, duration of HCQ use prior to conception, prophylactic low-molecular-weight heparin, low-dose aspirin, progesterone, and prednisone.

Ab, antibody; HCQ, hydroxychloroquine; OR, odds ratio; WG, week of gestation.

## Discussion

In our cohort of pregnancies in women with a history of RPL exposed to HCQ early in pregnancy, we found that the only factor determining the success of these pregnancies was the number of previous miscarriages. Thus, the risk of miscarriages was 65% in women with five or six prior miscarriages, and almost 100% in those with more than seven prior miscarriages.

Aneuploidy of the embryo is known to be a major cause of sporadic spontaneous abortions and RPL; it can be confirmed by the genetic analysis of POCs (Ogasawara *et al.*, 2000; Lei *et al.*, 2022). Nevertheless, the prevalence of chromosomal abnormalities in POCs appears to be lower for RPL than for sporadic pregnancy loss, and is strongly associated with the number of previous miscarriages (Ogasawara *et al.*, 2000; Lei *et al.*, 2022). Published studies have frequently focused on women with ≥2–3 RPL, but detailed data for situations involving six or more previous miscarriages are scarce (Ogasawara *et al.*, 2000; Lei *et al.*, 2022). In a large cohort of 1309 patients with 2–20 consecutive first-trimester abortions before a subsequent pregnancy, Ogasawara *et al.* (2000) found that miscarriage rates increased with the number of previous spontaneous abortions: 32% (3 miscarriages), 37% (4 miscarriages), 49% (5 miscarriages), 64% (6 miscarriages), and over 70% for at least seven miscarriages. The frequency of normal embryonic karyotypes increases with the number of prior miscarriages, whereas the rate of abnormal karyotypes remains constant. Thus, only about 20% of pregnancies in women with RPL ended in miscarriage due to an abnormal embryonic karyotype, regardless of the number of previous pregnancy losses (Ogasawara *et al.*, 2000). Moreover, patients who experience a miscarriage with an aneuploid embryo seem to have a greater chance of subsequently achieving a live birth than those with a miscarriage involving an euploid embryo (Ogasawara *et al.*, 2000; Carp *et al.*, 2001).

Cytogenetic analyses of POC samples are difficult to perform in practice, as miscarriages are often spontaneous, pass naturally, and frequently occur outside of clinical settings. In our study, a few POC analyses were performed in cases of early miscarriage (in women with four or five previous miscarriages), and no embryonic chromosomal abnormalities were found in 80% of these miscarriages. Advanced maternal age is a well-known independent factor associated with aneuploidy in the fetus (Franasiak *et al.*, 2014). Other parameters, such as diminished ovarian reserve (Jaswa *et al.*, 2021), abnormal parental karyotype (Klimczak *et al.*, 2021), or a high index of sperm DNA fragmentation (Klimczak *et al.*, 2021), may also be associated with a higher risk of aneuploidy in the embryo. In our study, none of these factors, including older age, was significantly more frequent in the group of pregnancies ending before 12 WG.

Thus, a large proportion of RPL remains unexplained by genetic causes, and current research aims to understand the other mechanisms involved, to facilitate the development of effective therapeutic interventions. Many factors and conditions have been identified as associated with recurrent miscarriages: behavioral factors, hormonal and metabolic diseases, uterine abnormalities, thrombophilia, and spermatic factors (Dimitriadis *et al.*, 2020; Quenby *et al.*, 2021; ESHRE Guideline Group on RPL *et al.*, 2023). However, the efficacy of specific treatments targeting these factors is often limited or unproven, and a direct causal link between these factors and RPL has yet to be clearly established (Dimitriadis *et al.*, 2020; ESHRE Guideline Group on RPL *et al.*, 2023). Dysregulation of the maternal immune system is increasingly recognized as a key factor underlying reproductive failure, as the balance between immune activation and tolerance within the endometrium is essential for successful pregnancy (Dimitriadis *et al.*, 2020; Colamatteo *et al.*, 2023). It has, therefore, been suggested that the immunomodulatory effects of HCQ, together with its anti-thrombotic, vascular-protective, and anti-infectious properties, might be beneficial for protecting against the mechanisms underlying unexplained RPL (de Moreuil *et al.*, 2020; Schrezenmeier and Dörner, 2020). We found that the incidence of early pregnancy loss increased with the number of previous miscarriages in women treated with HCQ, consistent with the findings for large cohorts (Ogasawara *et al.*, 2000; Lund *et al.*, 2012). However, in women with more than seven previous miscarriages, the proportion of pregnancies ending within the first trimester exceeded 90%. This suggests that HCQ may not be effective against causes of RPL other than embryonic chromosomal abnormalities, particularly those involving the maternal immune system.

A few studies are currently investigating the impact of HCQ on pregnancy outcomes in women with RPL, but published data are limited. Moini *et al.* (2022) studied 29 pregnant women with a history of unexplained RPL (≥2) receiving either HCQ (400 mg/d) or a placebo from early gestation (before 6 WG) to 20 WG. The miscarriage rate was 7.69% in the HCQ group and 28.57% in the placebo group, but the adjusted OR (2.96, 95% CI: 0.91–10.02) revealed no statistically significant difference after accounting for potential confounders (Moini *et al.*, 2022). The BBQ (HCQ for prevention of recurrent miscarriage) study is a randomized controlled trial designed to assess the potential impact of HCQ on live birth rates in women with at least three consecutive previous first-trimester miscarriages of unknown origin (Pasquier *et al.*, 2019). Treatment with a daily dose of 400 mg HCQ (or placebo) was initiated before conception and will be discontinued after 10 WG or earlier in case of pregnancy loss. The data from this study have not yet been published (Pasquier *et al.*, 2019). Finally, the ILIFE (immunosuppressant regimens for living fetuses) trial is an ongoing randomized controlled trial assessing the impact of HCQ, low-dose prednisone, and anticoagulation on the prevention of RPL in women with undifferentiated connective tissue disease (Yang *et al.*, 2020).

HCQ can have beneficial effects on obstetric APS by inhibiting the negative impact of aPL on trophoblast cell function, and by promoting vascular remodeling and placental development (de Moreuil *et al.*, 2020; Hoxha *et al.*, 2022). HCQ may, therefore, be considered an additional therapeutic option, in combination with LMWH and LDA, for preventing RPL in patients with APS (de Moreuil *et al.*, 2020; Hoxha *et al.*, 2022). Abisror *et al.* (2020) conducted a retrospective study in Europe comparing 187 women with at least one non-conventional aPL but without conventional aPL (seronegative APS) with 285 patients with conventional aPL (seropositive APS). Women with seronegative APS had significantly higher rates of miscarriages than those with seropositive APS (35% vs 6%) (Abisror *et al.*, 2020). In our study, non-conventional aPL were also frequently detected, but their distribution did not differ significantly between pregnancies ending before 12 WG and those progressing beyond this time point.

The lack of efficacy of HCQ in preventing miscarriage in our cohort could be related to the relatively short duration of HCQ exposure prior to conception. The pharmacokinetics of HCQ are complex, with significant variations in blood levels in treated humans, due to its large volume of distribution, high tissue binding, and long elimination half-life (Munster *et al.*, 2002; Schrezenmeier and Dörner, 2020; Dima *et al.*, 2022). Typically, steady-state concentrations are reached within 3–4 months, which may explain the delayed therapeutic response observed in autoimmune diseases such as systemic lupus erythematosus and rheumatoid arthritis (Schrezenmeier and Dörner, 2020; Dima *et al.*, 2022). In patients with these diseases, the clinical efficacy of HCQ is correlated with its blood concentration (Schrezenmeier and Dörner, 2020; Dima *et al.*, 2022). Munster *et al.* (2002) have shown that target concentrations in the therapeutic range (i.e. 1000 ng/ml) can be achieved after 5–6 weeks of weekly treatment at 400 mg per day, but there is considerable inter-individual variability. In the context of RPL, limited data on HCQ make it difficult to determine an optimal concentration to prevent the risk of further miscarriage. In our study, pregnancies were no longer viable >12 WG when HCQ was introduced before conception. Moreover, pregnancies exposed to HCQ for several months (>3months) did not have a lower incidence of new miscarriage than pregnancies exposed for a shorter duration. However, blood levels of HCQ were not measured during pregnancy or miscarriage.

Our study has several limitations. First, it did not include a control group. However, we report results for a large cohort of women with a large number of previous recurrent miscarriages exposed to HCQ during pregnancy, and almost no such data are currently available in the literature. We provide indirect evidence suggesting that HCQ has no significant efficacy against the immunological or vascular mechanisms potentially underlying these RPLs. In addition, not all patients had complete data for the diagnostic procedure for RPL. Furthermore, the results of endometrial biopsies investigating chronic endometritis and information about paternal age and behavioral factors were not available. It was, therefore, not possible to consider all the possible confounding factors. For pregnancies treated with HCQ in the post-conceptional period, the exact start date remains uncertain, as women should begin taking HCQ after a positive pregnancy test, ideally before 6 WG. Finally, our study is limited by the lack of available data on patient adherence and the absence of monitoring of HCQ blood levels.

## Conclusion

We found that the number of previous miscarriages was the only factor significantly associated with pregnancy progressing beyond the first trimester. The treatment of women with large numbers of repeated miscarriages with HCQ in early pregnancy does not appear to prevent the occurrence of further miscarriages.

## Data Availability

The data that support the findings of this study are available on request from the corresponding author. The data are not publicly available due to privacy or ethical restrictions. Data regarding any of the subjects in the study have not been previously published.
